# Circulating-tumour DNA methylation of *HAND1* gene: a promising biomarker in early detection of colorectal cancer

**DOI:** 10.1186/s12920-024-01893-9

**Published:** 2024-04-30

**Authors:** Mehrdad Shavali, Arash Moradi, Mohammad Tahmaseb, Kamal Mohammadian, Shahla Mohammad Ganji

**Affiliations:** 1 Department of Cell and Molecular Biology, Faculty of Biological Science, Kharazmi University, Tehran, Iran; 2 Department of Medical Biotechnology, National Institute of Genetic Engineering and Biotechnology, Shahrak-e Pajoohesh, km 15, P.O. Box 14965/161, Tehran, Tehran - Karaj Highway Iran; 3 Department of Radiation Oncology, Hamadan University of Medical Sciences, Hamadan, Iran; 4 Cancer Research Center, Hamadan University of Medical Sciences, Hamadan, Iran

**Keywords:** Methylation, Colorectal Cancer, ctDNA, *HAND1*, *SEPT9*

## Abstract

**Background:**

Colorectal cancer (CRC) is one of the significant global health concerns with an increase in cases. Regular screening tests are crucial for early detection as it is often asymptomatic in the initial stages. Liquid biopsies, a non-invasive approach that examines biomarkers in biofluids, offer a promising future in diagnosing and screening cancer. Circulating-tumour DNA (ctDNA) is the genetic material in biofluids released into the circulatory system by cells. ctDNA is a promising marker for monitoring patients since cancer cells display distinct DNA methylation patterns compared to normal cells. The potential of our research to contribute to early detection and improved patient outcomes is significant.

**Aims:**

The primary objective of this research project was to explore the *HAND1* methylation levels in plasma ctDNA as a potential biomarker for diagnosing CRC and evaluate the methylation level of the well-established gene *SPET9* to compare it with the methylation level of *HAND1*.

**Materials and methods:**

Plasma samples were collected from 30 CRC patients and 15 healthy individuals, with CRC samples obtained pre-treatment. ctDNA was extracted and treated with bisulfite for methylation status assessment. Quantitative methylation-specific PCR (qMS-PCR) was performed for *HAND1* and *SEPT9*, using β-actin (*ACTB* gene) as a reference. The study aims to evaluate the potential of these genes as diagnostic biomarkers for CRC, contributing to early detection and improved patient outcomes.

**Results:**

Our study yielded significant results: 90% of CRC patients (27 out of 30) had hypermethylation in the *SEPT9* gene, and 83% (25 out of 30) exhibited hypermethylation in the *HAND1* gene. The methylation levels of both genes were significantly higher in CRC patients than in healthy donors. These findings underscore the potential of *SEPT9* and *HAND1* methylation as promising biomarkers for diagnosing CRC, potentially leading to early detection and improved patient outcomes.

**Conclusion:**

These findings highlight the potential of *SEPT9* and *HAND1* methylation as promising biomarkers for diagnosing CRC. However, further research and validation studies are needed to confirm these findings and to explore their clinical utility in CRC diagnosis and management.

## Introduction

Colorectal cancer (CRC) is a significant global health concern; unfortunately, incidence rates are anticipated to continue to rise in the coming years. In 2023, it is estimated that the United States will experience approximately 153,020 new cases of CRC and 52,550 deaths related to the disease [1]. Despite a decrease in overall incidence, there has been a concerning shift toward younger age at diagnosis, advanced stage at diagnosis, and left colon/rectum cases [2]. Regular screening tests are crucial in detecting and diagnosing CRC early, as it is often considered a “silent” disease with no noticeable symptoms in its initial stages [3]. It is possible for patients not to experience bleeding or abdominal pain until the cancer has progressed to advanced stages, which can make treatment and cure more difficult.

Preventing disease spread and minimising illness’s impact on individuals is critical. Liquid biopsies, a medical method that examines biomarkers in biofluids (including blood plasma, saliva, cerebrospinal fluid, and urine), hold great potential as a non-invasive approach to diagnosing and screening cancer [4]. Circulating-tumour DNA (ctDNA) is a genetic material present in biofluids and released into the circulatory system by cells through various mechanisms. The release of ctDNA can occur due to apoptotic, necrotic, or tumoural activity. These DNA fragments range from a few to several hundred base pairs [5]. For instance, stepwise epigenetic and genetic alterations occur during the initiation and progression of CRC. There is increasing evidence that cancer cells display distinct DNA methylation patterns compared to normal cells. Typically, DNA methylation levels are decreased within cancer cells in areas of low CpG density compared to healthy cells. Conversely, a specific subset of CpG islands exhibit hypermethylation in a cancer cell-specific manner [6]. Hence, extracting valuable genetic information from tumour ctDNA could be crucial for cancer diagnosis, treatment, and screening.

*HAND1*, a gene located at chromosome 5q33, plays a crucial role in differentiating trophoblast giant cells and cardiac morphogenesis during development. Its involvement in cell proliferation and differentiation of trophoblast and cardiomyocytes has been well-documented [7]. In several types of cancer, including colorectal, pancreatic, small cell lung, ovarian, thyroid, and melanoma, *HAND1* is downregulated and methylated [8]. This gene, epigenetically silenced in colon cancer, has been identified as a Polycomb target closely associated with ES cell differentiation. Intriguingly, ectopic expression of *HAND1* triggers terminal differentiation and impedes the growth, proliferation, and xenograft tumour formation of colorectal cancer cells [9]. However, the underlying mechanism of this phenomenon remains a challenge, necessitating further research on HAND1 as a potential tumour suppressor in various cancer types. The possible implications of this research on cancer treatment underscore the importance of our collective efforts in this field.

ctDNA could be a promising marker for selecting and monitoring patients using the “watch and wait” approach. It is detected in about 75% of patients with CRC at the baseline and about 15–20% in the post-neoadjuvant or postoperative setting [10]. The primary objective of this research project was to explore the potential of methylation levels of well-known Septin-9 (*SEPT9*) and introduce a novel gene, Heart and Neural Crest Derivatives Expressed 1 (*HAND1*), in plasma ctDNA as the putative biomarker for diagnosing CRC. Specifically, this study aimed to investigate the methylation pattern of *HAND1* in ctDNA plasma samples for the first time. We utilised quantitative methylation-specific real-time PCR (qMS-PCR) to evaluate the levels of hypermethylation of *HAND1* and SEPT9 in ctDNA plasma samples obtained from Iranian patients diagnosed with CRC.

## Materials and methods

### Bioinformatic-based study of *SEPT9* and *HAND1* genes methylation level based on TCGA data

The *SEPT9* and *HAND1* promoter methylation status was analysed in Colorectal cancer using the UALCAN database (https://ualcan.path.uab.edu/). To do so, we obtained the data of Colon adenocarcinoma (COAD) patients, including Normal (*n* = 37), Stage1 (*n* = 50), Stage2 (*n* = 122), Stage3 (*n* = 88), and Stage4 (*n* = 41) patients data. UALCAN is a user-friendly and interactive web resource that comprehensively analyses cancer OMICS data, such as TCGA [11].

### Study design

In our previous bioinformatics-based study [12], we examined the areas where differential methylation in tissues affected by colorectal cancer (CRC). Our analysis uncovered a group of genes downregulated in CRC tissues due to hypermethylation. Within this group of genes, *HAND1* was one of the genes that showed significant hypermethylation and downregulation. Consequently, in the present study, we elected to scrutinise the methylation status of two genes, specifically *SEPT9* and HAND1, within the plasma of CRC patients. Our earlier discovery informed this decision about the HAND1 gene. The study design and methodology were carefully planned to ensure the reliability and validity of our findings.

### Patient and sample collection

Plasma samples from 30 patients with CRC and 15 healthy individuals were obtained from those referred to Besat Hospital in Hamedan Province, Iran, during January and March 2022. Healthy donors were checked for the absence of inflammation. Also, the samples of patients with CRC were collected before starting treatment (surgery or chemotherapy). Plasma samples were stored in the liquid nitrogen at -70˚C up to date of use for further analysis. The permission to conduct the present investigation was obtained from the Ethical Committee of NIGEB (Ethical Code: IR.NIGEB.EC.1401.12.14.D), and informed consent was obtained from patients.

### ctDNA extraction, bisulfite conversion, qMS-PCR

ctDNA was extracted from all the plasma samples using the standard protocol of the AddPrep Genomic DNA Extraction Kit (add Bio-Korea). Nanodrop evaluated the number of ctDNA. For assessing methylation status, EZ DNA Methylation-lightning TMKit (Zymo research-US) was used to treat 500ng of the isolated DNA with bisulfite, based on the manufacturer’s instruction.

This study aims to evaluate methylation status by Quantitative methylation-specific PCR (qMS-PCR) performed for *HAND1* and *SEPT9*, and β-actin (*ACTB* gene) as reference genes. The specific primers for methylated and unmethylated genes are designed as listed in Table 1. Bisulfite-treated HCT116 cell line (NCBIcode: C570) was used as the positive control, and ultrapure water was used as the control negative. The analysis of qMS-PCR was performed by utilising the MIC machine, USA, and SYBR Green reagents, which were triplicated on each sample. ACTB was selected as an endogenous reference gene in this analysis. qMS-PCR was performed using 1 µl of ctDNA (at a concentration of 50 ng/ µl), 0.3 µl of each forward and reverse primer, 6 µl of Hotstart RealQ Plus 2x Master Mix Green Without ROX (Amplicon, Denmark) and 4.4 µl of water in final of 12 µl. PCR amplification was run for these genes at two steps, with 1 of 95 °C for 5 min followed by step 2 composed of 40 cycles of 94 °C 30 s, 58 °C 45 s, and final 72 °C for 5 min extension. The analysis of methylation marker abundance of studied genes was calculated using REST 2009 software and according to the formula: 2^−(CtMarker−CtACTB) *100^.

**Table 1:** Sequences of the designed primers. MET, Methylated; UMET, unmethylated

Gene	Forward primer	Reverse primer	PCR production length
SEPT9-MET	5’- TAGTTAGCGCGTAGGGTTCGG − 3’	5’- AACCCAACACCCACCTTCG − 3’	**151 bp**
SEPT9-UMET	5’- TGTGGTGTTTTAGTTAGTGTG − 3’	5’- AATAATCCCATCCAACTACAC − 3’	**134 bp**
HAND1-MET	5’- GGTTTTTTAAAGATATCGGTTTC − 3’	5’- TTACCTATACGAACCTCGC − 3’	**114 bp**
HAND1-UMET	5’**-** GTTTTTTAAAGATATTGGTTTTG − 3’	5’**-** ATACCAAACATCACATTTAC − 3’	**129 bp**
ACTB-MET	5’**-** TCGGAAGTGGTTAGGGCGG − 3’	5’**-** GAAACCGACCTTACACATACCG − 3’	**128 bp**

### Data analysis

Statistical analysis was performed using GraphPad Prism 9.3.1. The Kolmogorov evaluated the normality and parameters of data using the Smirnov test. The expression levels of the candidate genes and patient’s demographics and clinical features, in addition to pathological risk factors, were tested by independent-sample *t*-test and one-way ANOVA, including Bonferroni and Tukey HSD tests following the assessment of the homogeneity of variances through the application of Levene’s test. The 95% confidence interval was used in all tests, and the *p*-value < 0.05 was considered significant. Mann–Whitney Plasma methylation levels between CRC cases and controls were compared using the U test to determine statistical significance. The sensitivity and specificity of the assay were evaluated using a receiver operating characteristic (ROC) curve, and the area under the ROC curve (AUC) was determined using a non-parametric method. The sensitivity of the methylation assay was calculated by determining what percentage of stage III-IV patients tested positive, and the specificity was calculated by determining what percentage of healthy controls tested negative.

## Results

### *SEPT9* and *HAND1* genes methylation status

**Fig. 1:**
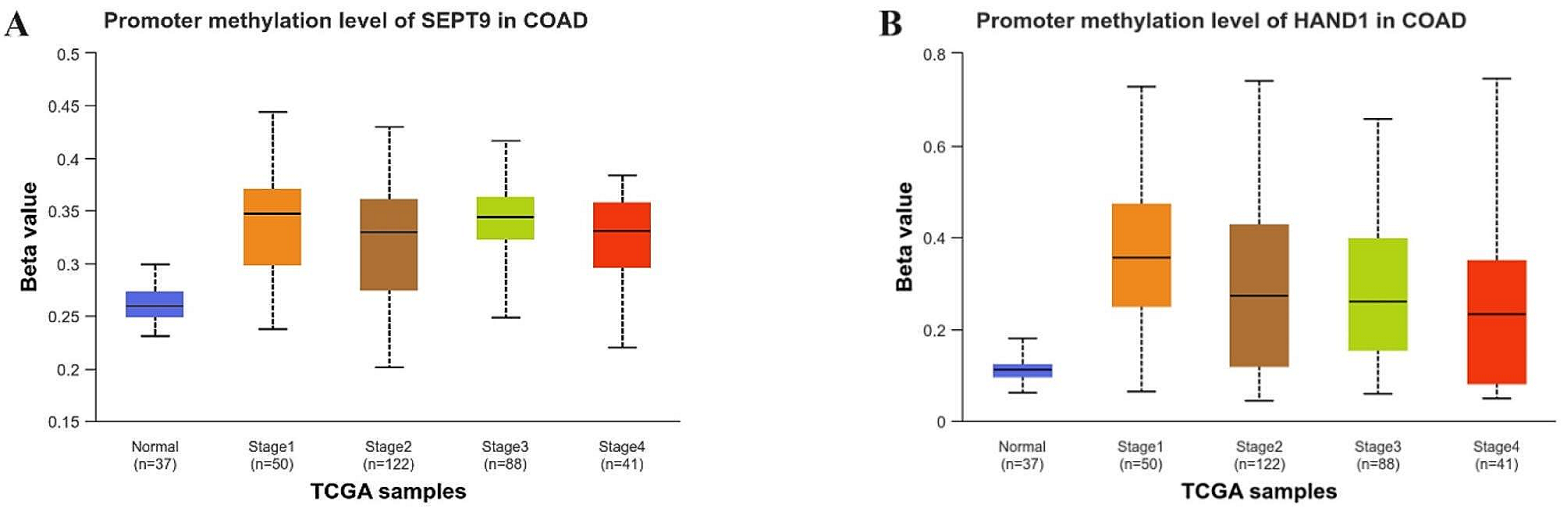
Promoter methylation levels based on TCGA data. The methylation levels of (**A**) *SEPT9* and (**B**) *HAND1* genes. Https://ualcan.path.uab.edu/ provides charts. COAD, Colon Adenocarcinoma

The data analysis reveals an interesting finding regarding the promoter *SEP9* gene, indicating a significant difference in methylation status across different stages of COAD when compared to the normal group. Specifically, the p-value of *SEP9* gene expression was found to be 1.63E-12 for Stage1, <1E-12 for Stage2, 1.62E-12 for Stage3, and 1.11E-10 for Stage4 (Fig. 1-A). Similarly, the promoter of the *HAND1* gene also showed a significant methylation alteration across the stages of COAD compared to the normal group. Specifically, the p-value of HAND1 gene expression was found to be 1.85E-13 for Stage1, < 1.62E-12 for Stage2, 1.62E-12 for Stage3, and 2.43E-05 for Stage4 (Fig. 1-B).

### Patient and sample characteristics

**Fig. 2:**
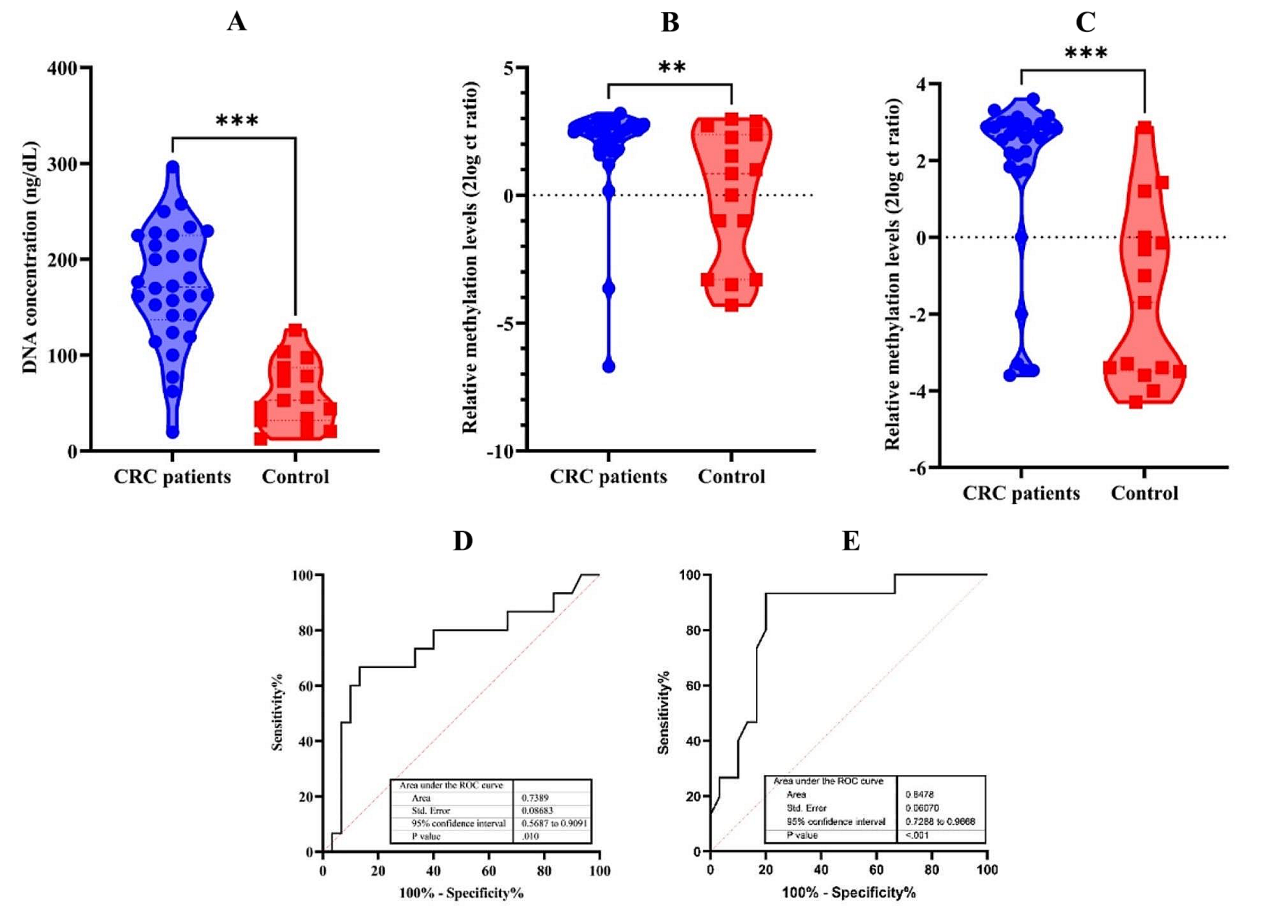
Concentration of extracted ctDNA from plasma samples and the relative methylation levels of genes. (**A**) ctDNA concentrations in CRC patients compared to normal controls. The mean ± SD of ctDNA concentrations in CRC patients was 171.90 ± 62.34, which is significantly (*p* < 0.001) higher than normal controls (58.79 ± 34.07). (**B**) Relative methylation levels of *SEPT9* (**, *p* = 0.009). (**C**) Relative methylation levels of *HAND1* (***, *p* < 0.001). (**D**) ROC curve for *SEPT9* (**E**) ROC curve for *HAND1*. ROC, Receiver-operating characteristics

The study comprised a group of patients diagnosed with CRC, consisting of 18 males (60%) and 12 females (40%), with an average age of 50.13 years and an age range of 31 to 68 years. On the other hand, the control group was composed of 8 males (54%) and 7 females (46%), with a slightly higher average age of 53.80 years and a wider age range of 29 to 72 years. The measured mean CRP levels in mg/dL were significantly higher (*p* < 0.001) in the CRC group, at approximately 23.08 mg/dL, compared to the control group. The concentrations of ctDNA in blood plasma were determined by microvolume spectrophotometer Nanodrop. The results conveyed that the concentrations of ctDNA in patients with CRC range from 19.62 to 296.31 ng/µL (median 171.90 ng/µL). The range of ctDNA concentration in healthy donors was significantly (*p* < 0.001) lower, from 12.68 to 126.01 ng/mL (median 58.79 ng/µL) (Table 2; Fig. 2-A). Due to the limitation in sample size, there was no significant correlation among the CRP, CA19-9, and CEA levels with ctDNA concentrations.

**Table 2:** Clinical data of CRC patients. CRP, C-reactive protein; CA 19 − 9, cancer antigen 19 − 9; CEA, Carcinoembryonic antigen; NA, Not applicable

Characteristic		CRC patients (*n* = 30)	Control (*n* = 15)	*P* value
**Sex**	**Male**	18 (60%)	8 (54%)	NA
	**Female**	12 (40%)	7 (46%)	NA
**Age**	**Mean ± SD**	50.13 ± 10.48	53.80 ± 14.02	0.324
	**Range**	31–68	29–72	NA
**Stage**	**T2N1**	9 (30%)	NA	NA
	**T3N1**	4 (13.3%)	NA	NA
	**T3N2**	2 (6.7%)	NA	NA
	**T4N0**	3 (10%)	NA	NA
	**T4N1**	3 (10%)	NA	NA
	**T4N2**	9 (30%)	NA	NA
**CRP (mg/dL)**		23.08 ± 9.62	1.23 ± 0.48	< 0.001
**CA 19 − 9 (U/mL)**		47.06 ± 8.33	NA	NA
**CEA (ng/mL)**		34.24 ± 17.82	NA	NA
**DNA concentration in plasma (ng/µL)**	**Mean ± SD**	171.90 ± 61.60	58.79 ± 34.07	< 0.001
	**Range**	19.62–296.31	12.68–126.01	NA

### ctDNA methylation levels of *SEPT9* and *HAND1* genes in plasma samples

Bisulfite treatment followed by quantitative methylation-specific polymerase chain reaction (qMS-PCR) was used to detect the methylation status of SEPT9 and HAND1 genes accurately. Our findings revealed that 90% of the samples from CRC patients (27 out of 30) had hypermethylation in the *SEPT9* gene, while 83% (25 out of 30) of the CRC patients exhibited hypermethylation in the *HAND1* gene. The methylation levels of the *SEPT9* gene were significantly (*p* = 0.009) higher in CRC patients (1.865 ± 2.053) than in healthy donors (0.0133 ± 2.591) (Fig. 2-B, D) with a p-value of 0.009. Notably, the methylation levels of the *SEPT9* CpG site in CRC patients showed a sensitivity of 66.67% (95% CI, 41.71–84.82%) and a specificity of 86.67% (95% CI, 70.32–94.69%), with a cut-off > 1.56. Similarly, the *HAND1* gene’s methylation status in CRC patients (1.617 ± 2.286) was significantly (*p* < 0.001) higher than in healthy donors (-1.545 ± 2.292) (Fig. 2-C, E). The methylation levels of the *HAND1* CpG site in CRC patients showed a sensitivity of 93.33% (95% CI, 70.18–99.66%) and a specificity of 80.00% (95% CI, 62.69–90.49%), with a cut-off > 1.57.

## Discussion

Tumour diagnosis is conventionally carried out by examining tissues through biopsy, considered the most reliable method. However, there are several limitations to this approach. For example, it is ineffective in detecting early-stage tumours or residual lesions. Moreover, this technique has limited application in assessing the effectiveness of treatment and predicting the prognosis of the disease. One promising biomarker that can be used to detect malignancy is circulating tumour DNA (ctDNA) found in the blood plasma. This biomarker can help overcome the limitations of tissue biopsy-based diagnosis and enable a more accurate and comprehensive cancer diagnosis [13]. Studies have shown that changes in ctDNA concentration can be linked to cancer patients’ development, prognosis, and survival [14]. The concentration of ctDNA tends to increase in patients with breast, gastric, ovarian, lung, colon, and prostate cancer, suggesting that it is associated with apoptosis and necrosis of cancer cells in the tumour microenvironment [15]. Researchers have also found specific cancer-related alterations in blood ctDNA, such as allelic imbalances, methylation, and mutations, which support this theory [16].

This study found that the concentration range of ctDNA in patients with CRC was between 19.62 and 296.31 ng/µL, with a median of 171.90 ng/µL. The concentration of ctDNA in healthy donors was significantly lower (*p* < 0.001), ranging from 12.68 to 126.01 ng/mL, with a median of 58.79 ng/µL. Previous research has revealed that the concentration of ctDNA in CRC patients can vary depending on the cancer stage and detection methods used. The range has been reported to be between 10 and 1000 ng/µL in some studies [17], while others have shown it to be between 0.1 and 12 ng/µL. Similarly, the ctDNA concentration in healthy donors has also varied in past studies, ranging from 0 to 100 ng/µL [18]. It is important to note that comparing results from different studies can be challenging due to differences in sample size, patient populations, and detection methods. Thus, elevated levels of ctDNA in patients’ plasma could be a biomarker for the presence of malignancy.

Recent studies have identified that the methylation of CPG islands in gene promoters, particularly *SEPT9*, could be used as a promising DNA biomarker for CRC diagnosis. SEPT9 is a member of the conserved family of cytoskeletal GTPases and is involved in various biological processes such as cytokinesis, polarisation, vesicle trafficking, membrane reconstruction, DNA repair, cell migration, and apoptosis [19]. Studies have shown that the *SEPT9* gene is regulated by a CpG island in its promoter region [19]. Methylation of the Spetin9 gene (^m^*SEPT9*) is the most extensively studied biomarker in CRC recurrence, and it is the only methylation-based biomarker approved for CRC diagnosis by the Food and Drug Administration (FDA) [20]. The methylated *SEPT9* (^m^*SEPT9*) has shown promise as a circulating biomarker for detecting colorectal cancer. In this study, we found that the methylation levels of SPET9 were significantly (*p* = 0.009) higher in CRC patients with a sensitivity of 66.67% (with a 95% confidence interval from 41.71 to 84.82%) and a specificity of 86.67% (with a 95% confidence interval from 70.32 to 94.69%), using a cut-off value of higher than 1.56. Aligned with our study, an investigation on ctDNA in CRC patients found that ^m^*SEPT9* was positive in 73% of CRC patients at 94.5% specificity. The sensitivity and specificity of ^m^*SEPT9* for diagnosis and recurrence monitoring were higher than that of CEA, CA19–9, and CA724 [21].

Furthermore, the methylation status of the *HAND1* gene in CRC patients was significantly higher (1.617 ± 2.286) compared to healthy donors (-1.545 ± 2.292) with a p-value of less than 0.001. Our study revealed that the methylation levels of the *HAND1* CpG site in CRC patients demonstrated a sensitivity of 93.33% (95% CI, 70.18–99.66%) and a specificity of 80.00% (95% CI, 62.69–90.49%), with a cut-off value greater than 1.57. It has been demonstrated that the expression of *HAND1* is controlled by the promoter region, which is regulated by cytosine methylation [8]. *HAND1*/2 proteins are essential in heart development, promoting cell proliferation and differentiation alongside other transcription factors like Nkx2-5 and GATA4. Researchers are interested in *HAND1*/2’s possible role as a tumour suppressor and how its downregulation may contribute to cardiomyopathy development. It is crucial to comprehend the multifaceted roles of these transcription factors in normal heart development and tumour formation [22]. Furthermore, studies demonstrated that the *HAND1* gene was hypermethylated and downregulated in gastric cancer [8] and is believed to function as a tumour suppressor gene. These findings suggest that *HAND1* gene methylation status may be a potential diagnostic biomarker for CRC patients.

In this study, we assessed the potential clinical usefulness of the *SPET9* and *HAND1* biomarkers by calculating their sensitivity and specificity. The bisulfite treatment followed by qMS-PCR was used to detect the methylation status of genes, which enhanced the study’s reliability. Nevertheless, the study had some limitations that require further attention. For example, the limited sample size must be expanded to achieve more statistically meaningful outcomes. Further longitudinal data on ctDNA concentrations or methylation status variations concerning disease progression or treatment response should be undertaken. On the other hand, there is a recommendation to conduct more investigations on dimension reduction approaches [23], which are critical to enhancing cancer research. This method can facilitate the selection of the most informative potential biomarkers that could impact the diagnostic precision. It is suggested that examining methylation alterations in non-cancerous conditions like polyps and inflammatory bowel disease (IBD) can differentiate between malignant and non-malignant diseases. Therefore, more research is crucial to validate these findings and comprehend their practicality in a clinical setting.

## Conclusion

In conclusion, the study’s findings provide valuable insights into the potential of *SEPT9* and *HAND1* methylation as highly promising biomarkers for diagnosing CRC. These epigenetic alterations have been shown to exhibit high sensitivity and specificity in differentiating CRC from non-cancerous tissues and other types of colorectal cancer. However, it is essential to note that further research and validation studies are necessary to corroborate these results and to assess their clinical usefulness in CRC diagnosis and management. The development of reliable biomarkers for CRC detection is crucial for improving early diagnosis and treatment outcomes, and it is hoped that the findings of this study will pave the way for more effective diagnostic tools and therapeutic strategies in the future.

## Data Availability

The data generated and/or analysed during the current study are not publicly available but are available from the corresponding author who organised the study.
